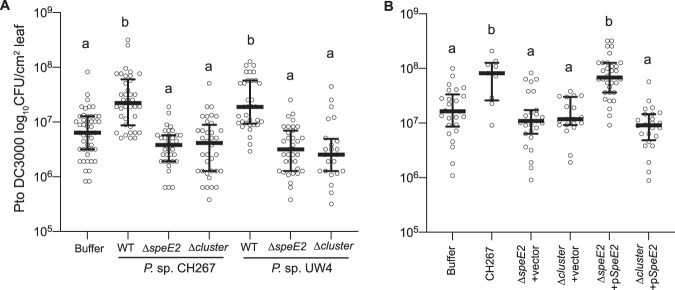# Erratum for Beskrovnaya et al., “Comparative Genomics Identified a Genetic Locus in Plant-Associated *Pseudomonas* spp. That Is Necessary for Induced Systemic Susceptibility”

**DOI:** 10.1128/mBio.01906-20

**Published:** 2020-08-11

**Authors:** Polina Beskrovnaya, Ryan A. Melnyk, Zhexian Liu, Yang Liu, Melanie A. Higgins, Yi Song, Katherine S. Ryan, Cara H. Haney

**Affiliations:** a Department of Microbiology and Immunology, The University of British Columbia, Vancouver, Canada; b Department of Chemistry, The University of British Columbia, Vancouver, Canada; c State Key Laboratory of Genetic Engineering and Fudan Institute of Plant Biology, School of Life Sciences, Fudan University, Shanghai, China; d Michael Smith Laboratories, The University of British Columbia, Vancouver, Canada

## ERRATUM

Volume 11, no. 3, e00575-20, 2020, https://doi.org/10.1128/mBio.00575-20. In Fig. 3B, the labels for the 3rd and 4th data sets previously labeled Δ*speE2*+pSpeE2 and Δcluster+vector, respectively, were swapped. The correctly labeled figure appears below. There are no corresponding changes to the text or figure legend.

**Figure 1:**